# Amerindian *Helicobacter pylori* Strains Go Extinct, as European Strains Expand Their Host Range

**DOI:** 10.1371/journal.pone.0003307

**Published:** 2008-10-02

**Authors:** Maria G. Domínguez-Bello, Maria E. Pérez, Maria C. Bortolini, Francisco M. Salzano, Luis R. Pericchi, Orlisbeth Zambrano-Guzmán, Bodo Linz

**Affiliations:** 1 Department of Biology, University of Puerto Rico, San Juan, Puerto Rico, United States of America; 2 Department of Mathematics, University of Puerto Rico, San Juan, Puerto Rico, United States of America; 3 Department of Genetics, Universidade Federal Rio Grande do Sul, Porto Alegre, Brazil; 4 Bilingual Intercultural Department, Universidad Pedagogica Experimental Libertador, Puerto Ayacucho, Venezuela; 5 Department of Molecular Biology, Max-Planck-Institut für Infektionsbiologie, Berlin, Germany; Oxford University, United Kingdom

## Abstract

We studied the diversity of bacteria and host in the *H. pylori*-human model. The human indigenous bacterium *H. pylori* diverged along with humans, into African, European, Asian and Amerindian groups. Of these, Amerindians have the least genetic diversity. Since niche diversity widens the sets of resources for colonizing species, we predicted that the Amerindian *H. pylori* strains would be the least diverse. We analyzed the multilocus sequence (7 housekeeping genes) of 131 strains: 19 cultured from Africans, 36 from Spanish, 11 from Koreans, 43 from Amerindians and 22 from South American Mestizos. We found that all strains that had been cultured from Africans were African strains (*hpAfrica1*), all from Spanish were European (*hpEurope*) and all from Koreans were *hspEAsia* but that Amerindians and Mestizos carried mixed strains: *hspAmerind* and *hpEurope* strains had been cultured from Amerindians and *hpEurope* and *hpAfrica1* were cultured from Mestizos. The least genetically diverse *H. pylori* strains were *hspAmerind*. Strains *hpEurope* were the most diverse and showed remarkable multilocus sequence mosaicism (indicating recombination). The lower genetic structure in *hpEurope* strains is consistent with colonization of a diversity of hosts. If diversity is important for the success of *H. pylori*, then the low diversity of Amerindian strains might be linked to their apparent tendency to disappear. This suggests that Amerindian strains may lack the needed diversity to survive the diversity brought by non-Amerindian hosts.

## Introduction

Humans coevolved with their microbiomes [1]–[3] and persistent human microbes can be markers of human migrations. Viruses such as HPV [4], Hepatitis G [5], RNA retrovirus HTLV-1 [6] and the bacterium *Helicobacter pylori* [7]–[9] show signs of coevolution with their human host, reflecting the serial founding African-origin model of human evolution due to successive human migrations that consisted of only a subset of the genetic variation available at the source location [10], [11].

**Figure 1:**
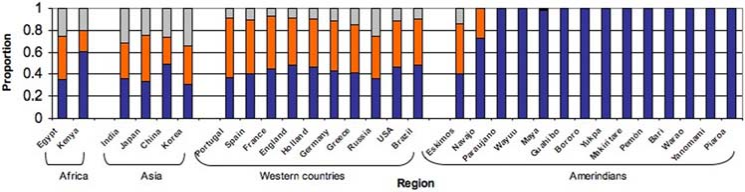
Distribution of ABO blood groups in humans from Africa, Asia, Western countries and in Amerindians. The blue, orange and grey bars represent respectively O, A and B allele frequencies [34]–[43]. The remarkable dominance of O blood groups among Amerindians from South America affects the ABO blood group recognition by *H. pylori* strains.

The original human migration out of Africa occurred approx. 60,000 years ago, towards the Middle East and thereafter independently to Europe and Asia [12]. The Americas were populated by humans of East Asian ancestry that crossed the Bering Strait, about 15 thousand years ago. These first Americans suffered a genetic bottleneck [13]–[15], and the reduced genetic diversity in Amerindians is evidenced in the absolute dominance of the O blood group among Amerindians (**Fig. 1**), their low heterozygosity and the reduced number of mitochondrial DNA haplogroups [16], [17]. However, the diversity of Amerindians has increased through genetic inflow from Europeans and Africans over the last 500 years, leading to an increasing Mestizo population.

*H. pylori* is a human gastric indigenous bacteria, in spite of being a risk factor for gastric cancer and peptic ulcer disease [18], [19]). A congruent divergence of *H. pylori* and their human hosts suggests that the quality of host resources influences the diversity of indigenous bacteria and their adaptations. In host-parasite interactions, a restricted gene flow in the host leads to local microbial adaptation [20], [21]. An example of local adaptation is the differing affinity of *H. pylori* strains to bind blood group antigens expressed in the human gastric mucosa: European *H. pylori* strains bind all three (A, B and O) blood group antigens, but an important proportion of strains from the are dominantly O blood group Amerindians have higher affinity for O blood antigen [22].

Environmental diversity allows different species to occupy different niches, sustaining coexistence and species diversity. There is a strong body of evidence for the loss of diversity by reduction of niches. In addition to landscape mosaicism, temporal variability also affects diversity. Simulations predict that while spatial variability increases diversity, the strongest increase occur at intermediate levels of temporal variability [23]. When the environment of a species is provided by another species, there is a set of age-changing mosaic niches provided by each individual host for the life of the host. Since microbes circulate in a dynamic population of hosts, the microbe diversity is held at the host population –and not individual- level. The studies that have provided the basis for many ecology theories have come from the field of plant ecology. Plant ecological studies have shown that diversity of plant associated herbivores increases with diversity of plant species [24] but also with population genotypic diversity, within a single species [25]. As such, a direct association between diversities in host and microbiome microbes could be expected. To test this hypothesis, we determined intra-population genetic diversity of strains of the highly diverse gastric bacteria *H. pylori* and of humans from the Americas and other geographical populations relevant to its peopling.

## Results

We determined the intra-population genetic diversity of multilocus sequences of 7 housekeeping genes from 131 strains of the gastric bacteria *H. pylori* and of 2,232 sequences of the human mtDNA hypervariable segment I. *H. pylori* strains were selected from human populations relevant to the ancestry of the people of the Americas, with East Asians providing a reference group for Amerindians: 131 *H. pylori* isolates were from Amerindians, Spanish, West-Africans, South American Mestizos and Koreans. Based on their DNA multilocus sequences, the strains were assigned to one of 6 geographical bacterial populations: *hpEastAsia* (subpopulations *hspAmerind*, *hspMaori* and *hspEAsia*), *hpAsia2*, *hpEurope*, *hpNEAfrica*, *hpAfrica1* (*hspWAfrica*, *hspSAfrica*) and *hpAfrica2* [7], [26]. Selected human sequences were from similar locations to those where *H. pylori* strains had been isolated. A total of 1,148 sequences of human *mtDNA* hypervariable segment I from non-Bantu Africans, Spanish, Koreans, Amerindians (Guahibo, Huitoto, Inuit) and Latin American Mestizos were retrieved from Genbank and from published work. Bacterial and human genetic distance matrixes were generated and compared using non-parametric statistics.

### Strain genetic diversity

**Figure 2:**
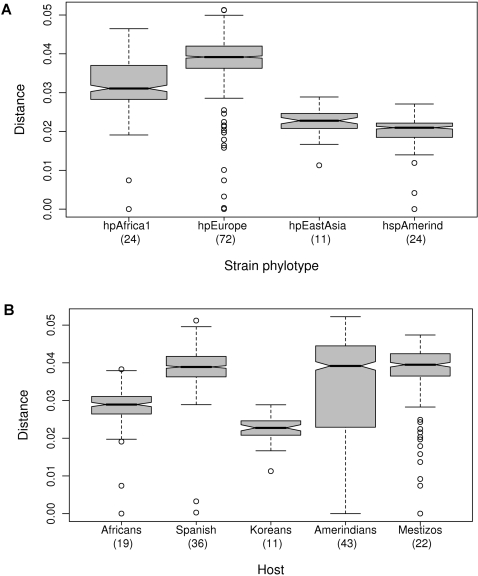
Pairwise genetic distances between *H. pylori* strains grouped by bacterial population (2A), or according to their human host (2B). Differences in pairwise distances among strains in Fig. 2A were significant (Kruskal-Wallis test, p<2.2×10^−16^, Wilcoxon and Bonferroni; p<10^−14^) with a decreasing order *hpEurope*>*hpAfrica1*>*hpEastAsia*>*hspAmerind*. When grouped by the human host from which each strain was isolated (Fig 2B), strain diversity in Amerindians was as high as in Spanish and Mestizos (with no significant differences among them; Wilcoxon Pairwise comparison *p*>0.7), with a decreasing order Spaniards = Amerindians = Mestizos>Africans>Koreans. The waist of the dress-like box is the median with the waist side openings indicating the 95% interval for the median; the top represents the 3rd quartile and the bottom the 1st quartile. The interval in dashed lines represents a maximum of 1.5× interquartile range and the open circles are outliers. Permutation tests confirmed group differences in strain diversity.

**Figure 3:**
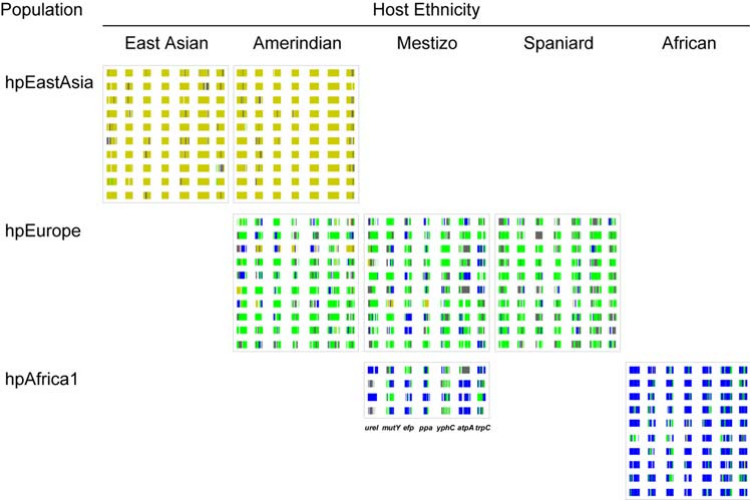
Mosaic structure of the multilocus *H. pylori* sequences in representative strains. The ancestral source of each polymorphic nucleotide is shown by a vertical line for each of the seven gene fragments in the multilocus analysis of 10 representative strains from each group (see the legend below Mestizo strains). Individual nucleotides were derived from ancestral Europe1 (grey), ancestral Europe2 (green), ancestral Africa1 (blue) and ancestral EastAsia (yellow). Nucleotides not assigned with >50% probability to any one population are indicated by white lines. African and European components can be observed in *hpEurope* strains from Spaniards and Mestizos, as well as in *hpAfrica1* from Mestizos, while African *hpAfrica1* strains and Amerindian *hspAmerind* strains tested were largely homogeneous.

The bacterial strains were assigned to populations according to their multilocus DNA sequences: those from African hosts yielded only *hpAfrica1*, those from Spanish yielded *hpEurope* and those from Koreans yielded *hspEAsia* (**Table 1**). However, Huitoto and Guahibo Amerindians yielded both *hspAmerind* and *hpEurope* strains, and Mestizos yielded *hpEurope* and *hpAfrica1*, but not *hspAmerind*. The least and most diverse strains of *H. pylori* populations were *hspAmerind* and *hpEurope*, respectively (**Figure 2A**). Nonetheless, when grouping the strains by host, strain diversity in Amerindians increased to the levels found in Spanish and Mestizo hosts (**Figure 2B**), consistently with the circulation of mixed strains (**Table 1**) and with the remarkable mosaicism reflected in the multilocus sequences (**Figure 3** **)**. As stated previously [7], [26], the ancestry patterns of modern *hpEurope* strains revealed extensive recombination between the two ancestral populations ancestral Europe1 and ancestral Europe2. Spanish *H. pylori* (**Figure 3** **)** further include components from ancestral *hpAfrica1*, which possibly reflects the role Africa has played in shaping the Spanish modern human gene pool. Traces of African bacterial ancestry were also detected in strains from Mestizos and Amerindians. The observed mosaicism reflects extensive recombination and results in lower genetic structure.

**Table 1:** *H. pylori* assignment to bacterial populations.

Location/source of human population	Bacterial population				
	No. of strains	*hpAfrica1*	*hpEurope*	*hpEAsia*	*hspAmerind*
**African**	**19**	**19**			
Senegal	5	5			
Burkina Faso	14	14			
**Europe**	**36**		**36**		
Spain	36		36		
**East Asian**	**11**			**11**	
Korea	11			11	
**Amerindian**	**43**		**19**		**24**
Inuit (Eskimo)	13		4		9
Athabaskan (Na-Dene)	6				6
Huitoto	16		12		4
Piaroa	3				3
Guahibo	5		3		2
**Mestizo**	**22**	**5**	**17**		
Colombia	12	1	11		
Venezuela	10	4	6		
All	131	24	72	11	24

Multilocus sequences of the strains were from 7 housekeeping genes (*atpA*, *efp*, *mutY*, *ppa*, *trpC*, *ureI* and *yphC*). While only the expected populations were identified in African, Spanish and Korean hosts, strains assigned to more than one population were observed among Amerindians and Mestizos.

### Human genetic diversity

Host genetic diversity increased in this study in the order: Amerindians<Spaniards<Koreans<Africans<Mestizos (Kruskal-Wallis *p*<10^−14^, **Figure S4**). However, measurement of human diversity through mtDNA underestimates paternal-linked diversity. Mestizos diversity has probably been introduced by both maternal (Amerindian and African) and paternal (European and African) gene flow, but the remarkably low diversity in the Spanish mtDNA may not reflect the true variability of human genetic diversity introduced in Spain by paternal lines.

## Discussion

Human evolution has been shaped by the early forms of life in the planet, the microbes. Indeed, evolution of all eukaryotes occurred in a bacterial planet, and microbes found niches on surfaces, invaginations and guts of humans, as well. Helicobacters found a niche in the gastric pouch of mammals [27], including humans. *Helicobacter pylori* has colonized the human stomach since the beginning of the human history [26], and maintained a tuned evolution with its host, reflecting in its genome some genetic, linguistic and cultural traits of the human population. It can therefore be a useful model to determine how humans modulate the diversity of their microbiomes.

### Genetic divergence and convergence

Although most of the data analyzed in this study was already available, they had previously not been analyzed in terms of the associations between genetic diversity of humans and their indigenous microbial population. There are many genetic studies that confirm that human intrapopulation genetic diversity decreases with geographic distance from Africa, and that the Amerindians suffered a genetic bottleneck [12]–[17]. The finding that a low-diversity human population was associated with a low-diversity bacterium could be due to increased genetic drift (on a populations of low effective size), and/or selection by low diversity blood groups on the *H. pylori* population.

Genetic isolation and drift as well as selection may explain the radiation of humans into different groups, and the concomitant divergence of *H. pylori* into geographical types, whereby both host and bacteria show patterns of isolation by distance [26]. However, the modern world is increasingly bringing genetic influx into each of the human groups, both at the human and microbiome level. Indigenous microbes that once diverged during most of human history are currently in the process of converging, which erase phylogeographic signals. How this process is occurring is important because some human-evolved bacteria are relevant to human health (for example *H. pylori* is implicated in gastric diseases), and changes in the dynamics of its coevolution with humans might lead to changes in disease patterns.

### Genetic diversity and fitness

There might be greater local genetic variation in parasite populations relative to their hosts. In this case, parasite populations might increase their genetic structure by locally adapting to their hosts [28]. Consistently, mosaic strains would be expected to be genetically more diverse and more generalist. This might explain why *hpAfrica1* strains from Mestizos are more diverse than those from African hosts (Kruskal-Wallis p<10−14; **Figure S1**). Recombination obviously requires coexistence of different strains in a single stomach, which we indeed have found to be frequent in patients from Venezuela [29].

Diversity optimizes niche partitioning. In the gastric context, strain mosaicism (intra-genomic diversity) and strain diversity (inter-genomic variants) is likely to be a key factor in the *hpEurope* strains host range expansion (in both Mestizos and Amerindians). Meanwhile, *hspAmerind* strains seem to lack the diversity that would be required to survive in the high host variability brought by Mestizos. Displacement of *hspAmerind* by *hpEurope* strains could occur by two different means: **1) Strain competition** during colonization, in which “generalist” strains will have better chance to colonize diverse niches than “specialist” strains [as in the case of O binder Amerindian strains described above, which would have less success in colonizing non-O blood group Mestizos [22]]; **2) Strain subversion by transformation**, in which DNA from *hpEurope* strains is taken up and recombinant strains become increasingly European. The high component of European ancestry in the mosaic strains from Mestizos and Amerindians, and the relatively low Amerindian component in Mestizos (**Figure 3**) support this second hypothesis.

**Figure 4:**
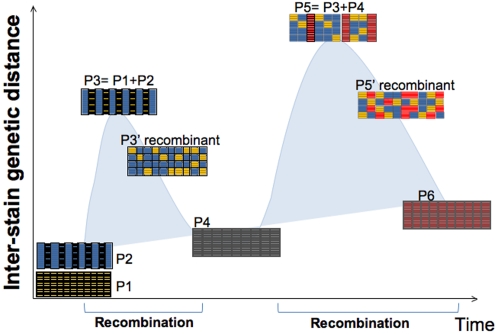
Development of inter-strain genetic diversity over time as two populations recombine. In the context of our hypothesis, a low diversity *H. pylori* population (P1) arose from co-evolution with the isolated Amerindian host population. With the introduction of new *H. pylori* strains (P2) the new population formed (P3) is more diverse than any of the source populations alone. Selection acts and strains recombine with the consequent homogenization of the population (P3′). The cycle is repeated when new populations (P4) arrive. Given time and isolation (no gene flow), population diversity will be reduced (P6). Based on their mosaic structure and high genetic distances, it seems that current *H. pylori* from Amerindians and Mestizos are in one of the intermediate states (P3′ or P5′). Arrows indicate introduction of new populations.

The consumption of antibiotics and other drugs (such as proton pump inhibitors that reduce gastric acidity) might also be currently affecting the bacterial populations by increasing selection pressure in favor of particular variants, thus shaping the genome of indigenous microbes in modern humans. Modern human admixture results not only in increased human diversity but also in increased microbial diversity of the human microbiome. In the long term, however, one can predict that maximum genetic distances will be succeeded by homogenization of the genomic structure in strain variants (all mosaic or recombinant strains) and humans (all Mestizos) and decreased within population genetic distances brought by further recombination and lack of isolation, as illustrated in **Figure 4**. Since no influx of genetic variation is expected from outside the global village, low genetic flow in both microbes and hosts will eventually result in decreased human and microbiome diversity.

To our knowledge, Wirth et al. [30] were the first to analyze both human DNA and *H. pylori* sequences from 50 patients in Ladakh in northern India. Our results support the need of a study with a true large scale population-based approach involving human and microbe samples from the same hosts in order to better measure the extent in which human populations shape the genomes of *H. pylori*. As new technology optimizes cost and time of sequencing, there can be new studies performed in individual *Homo sapiens* and his individual microbiome, using more genes. These studies will be crucial to understand the coevolution of humans and their microbes.

### Conclusions

In the analysis of differences in genetic diversity in human groups and the concomitant diversity of a human indigenous microbe, our study provides support to the hypothesis that the distribution of genetic diversity in humans determines the genetic diversity of indigenous microbes. Specifically, we found genetic evidence of i) a decreased diversity of *H. pylori* strains in the human group with the least genomic diversity; ii) an increased diversity in strains from Mestizos, hybrid strains with mosaic structure assigned to the *hpEurope* group; iii) a host-range expansion of these *hpEurope* strains into Mestizos and Amerindian hosts, confirming that niche diversity widens the sets of resources for colonizing species.

## Materials and Methods

### *H. pylori* strains

We selected strains from Amerindians and other human populations relevant to the peopling of the Americas. These included, Spanish, West-Africans, Mestizos, and East Asians who provide a reference group for Amerindians.

We analyzed multilocus sequences from 7 housekeeping genes (*atpA*, *efp*, *mutY*, *ppa*, *trpC*, *ureI* and *yphC*) [7] in 131 *H. pylori* isolates cultured from 19 Africans (14 from Burkina Fasso, 5 from Senegal), 36 Spanish, 11 Koreans, 43 Amerindians (13 Inuit, 6 Athabaskan, 16 Huitoto, 5 Guahibo and 3 Piaroa), and 22 Mestizos (12 from Colombia and 10 from Venezuela). Sequences were available in http://www.mlst.net/ and the EMBL database except for 14 sequences experimentally obtained in this work, from 8 Amerindians (5 Guahibos, 3 Piaroas) and 6 Venezuelan Mestizos, now available in GenBank (accession numbers EU878038–EU878135) and in the MLST database in Oxford (http://www.mlst.net/).

Strain population assignment was performed as described by Falush et al [7], using the “no admixture” model in STRUCTURE2.0 [31], the proportion of nucleotides being derived from ancestral population was estimated using the “linkage” model in Structure2.0 as described [7], [26].

### Human *mtDNA* sequences

A total of 1,148 sequences of human *mtDNA* hypervariable segment I were retrieved from Genbank from published work (Table 2), from humans close to the hosts from which *H. pylori* strains had been cultured: non-Bantu Africans, Spanish, Koreans, Amerindians (Guahibo, Huitoto, Inuit) and South American Mestizos.

**Table 2:** Sources of human *mtDNA* sequences analyzed in this study.

Host group	Number of mtDNA sequences	References
**Africans**	53	[44]
Hausa (non-Bantu)	13	
Sierra Leone (non-Bantu)	40	
**Europeans-Spanish**	718	
Andalusia	130	Genbank; [45]–[47]
North Eastern	118	[48]
Catalans	61	[45]–[47]
Baleares	222	[49]
Galicia	92	[50]
Canary Island,	54	[46]
Spain various	41	[46], [47]
**East Asians-Koreans**	64	[51]
**East Asians- Amerindians**	143	
Guahibo	66	GenBank
Huitoto	7	[52]
Inuit (Eskimo)	70	[53]
**Mestizos** a	170	[54]
African haplotypes	58	
European haplotypes	59	
Amerindian haplotypes	53	
**TOTAL**	1,148	

a From Venezuela and Colombia.

### Statistical analyses

Genetic distances (pairwise distances) of *H. pylori* isolates and human mtDNA were analyzed by strain ancestral phylogroups and by human host groups. Distances were calculated using the Kimura 2-parameter model in MEGA3 [32]. Nonparametric tests (Kruskal-Wallis rank sum, Wilcoxon test with Bonferroni adjustment for multiple comparisons) were used to compare strains by bacterial population and by host. In addition, these comparisons were performed using permutation tests based on 5000 permutations. All statistical analyses were performed using R statistical software [33].

## Supporting Information

Figure S1 Intrapopulation genetic distance of H. pylori strains hpAfrica1 and hpEurope, by host source. hpAfrica1 strains from Mestizos were much more diverse than those from Africans (Kruskal-Wallis p<10−14). In contrast, neither Mestizos nor Amerindians increased the already high variability hpEurope strains from Spain. Medians are represented as the waist of the dress-like box, and the waist side openings indicate the 95% interval for the median. Above and below the median are the 3rd and 1st quartile respectively. The interval in dashed lines represents a maximum of 1.5× interquartile range and the open circles are outliers. Outliers are mostly low pairwise distances, indicating that similar pair of strains are less common than distant strains.
(0.24 MB TIF)

Figure S2 Genetic distances between mtDNA sequences of diverse human groups. Differences between median distances were significant for each of the human groups (Kruskal-Wallis test p<2.2×10−15; Wilcoxon with Bonferroni adjustment p<10−14). Permutation tests with 5,000 permutations confirmed human group differences (p = 0). Distances decreased in the order: mestizos>Africans>Koreans>Spanish> Amerindians. For explanation of the box plot see Figure S1.
(18.39 MB DOC)

Figure S3 Genetic distances of human mtDNA sequences within Africans, Spanish, Koreans and groups of Amerindians and Mestizos. South American Amerindians studied have a degree of admixture as indicated by their higher genetic diversity than the Inuit. The Mestizos with African or Amerindian haplotypes have increased diversity in relation to Mestizos with European haplotypes. For explanation of the box plot see Figure S1.
(0.26 MB TIF)

## References

[1] Backhed F, Ley RE, Sonnenburg JL, Peterson DA, Gordon JI (2005). Host-bacterial mutualism in the human intestine.. Science.

[2] Eckburg PB, Bik EM, Bernstein CN, Purdom E, Dethlefsen L (2005). Diversity of the human intestinal microbial flora.. Science.

[3] Gao Z, Tseng CH, Pei Z, Blaser MJ (2007). Molecular analysis of human forearm superficial skin bacterial biota.. Proc Natl Acad Sci U S A.

[4] Ho L, Chan SY, Burk RD, Das BC, Fujinaga K (1993). The genetic drift of human papillomavirus type 16 is a means of reconstructing prehistoric viral spread and the movement of ancient human populations.. J Virol.

[5] Loureiro CL, Alonso R, Pacheco BA, Uzcategui MG, Villegas L (2002). High prevalence of GB virus C/hepatitis G virus genotype 3 among autochthonous Venezuelan populations.. J Med Virol.

[6] Miura T, Fukunaga T, Igarashi T, Yamashita M, Ido E (1994). Phylogenetic subtypes of human T-lymphotropic virus type I and their relations to the anthropological background.. Proc Natl Acad Sci U S A.

[7] Falush D, Wirth T, Linz B, Pritchard JKMS (2003). Traces of Human Migrations in *Helicobacter pylori* Populations.. Science.

[8] Wirth T, Meyer A, Achtman M (2005). Deciphering host migrations and origins by means of their microbes.. Mol Ecol.

[9] Covacci A, Telford JL, Del Giudice G, Parsonnet J, Rappuoli R (1999). Helicobacter pylori virulence and genetic geography.. Science.

[10] Ramachandran S, Deshpande O, Roseman CC, Rosenberg NA, Feldman MW (2005). Support from the relationship of genetic and geographic distance in human populations for a serial founder effect originating in Africa.. Proc Natl Acad Sci U S A.

[11] Prugnolle F, Manica A, Balloux F (2005). Geography predicts neutral genetic diversity of human populations.. Curr Biol.

[12] Cavalli-Sforza LL, Menozzi P, Piazza A (1994). The history and geography of human genes.

[13] Bortolini MC, Salzano FM, Thomas MG, Stuart S, Nasanen SP (2003). Y-chromosome evidence for differing ancient demographic histories in the Americas.. Am J Hum Genet.

[14] Reidla M, Kivisild T, Metspalu E, Kaldma K, Tambets K (2003). Origin and diffusion of mtDNA haplogroup X.. Am J Hum Genet.

[15] Schurr T, Sherry ST (2004). Mitocondrial DNA and Y chromosome diversity and the peopling of the Americas: evolutionary and demographic evidence.. Am J Hum Biol.

[16] Wang S, Lewis CM, Jakobson M, Ramachandran S, Ray N (2007). Genetic Variation and Population Structure in Native American.. PLoS Genet.

[17] Liu H, Prugnolle F, Manica A, Balloux F (2006). A geographically explicit genetic model of worldwide human-settlement history.. Am J Hum Genet.

[18] Parsonnet J (1994). Gastric adenocarcinoma and *Helicobacter pylori* infection.. West J Med.

[19] Blaser MJ, Atherton JC (2004). *Helicobacter pylori* persistence: biology and disease.. J Clin Invest.

[20] Gandon S (1998). Local adaptation and host-parasite interactions.. Trends Ecol Evol.

[21] Kirkpatrick M, Barton NH (1997). Evolution of a species range.. Am Nat.

[22] Boren T, Falk P, Roth KA, Larson G, Normark S (1993). Attachment of *Helicobacter pylori* to human gastric epithelium mediated by blood group antigens.. Science.

[23] Reineking B, Veste M, Wissel C, Huth A (2006). Environmental variability and allocation trade-offs maintain species diversity in a process-based mofel of succulent plant communities.. Ecological Modelling.

[24] Armbrecht I, Perfecto I, Vandermeer J (2004). Enigmatic biodiversity correlations: Ant diversity responds to diverse resources.. Science.

[25] Crutsinger GM, Collins MD, Fordyce JA, Gompert Z, Nice CC (2006). Plant genotypic diversity predicts community structure and governs an ecosystem process.. Sience.

[26] Linz B, Balloux F, Moodley Y, Manica A, Liu H (2007). An African origin for the intimate association between humans and *Helicobacter pylori*.. Nature.

[27] Domínguez-Bello MG, Blaser JM (2008). Evolution of Helicobacter and Helicobacter Infections.. Evolutionary Biology of Bacterial and Fungal Pathogens.

[28] Kaltz O, Shykoff JA (1998). Local adaptation in host-parasite systems.. Heredity.

[29] Ghose C, Perez-Perez GI, van Doorn LJ, Dominguez-Bello MG, Blaser MJ (2005). High frequency of gastric colonization with multiple *Helicobacter pylori* strains in Venezuelan subjects.. J Clin Microbiol.

[30] Wirth T, Wang X, Linz B, Novick RP, Lum JK (2004). Distinguishing human ethnic groups by means of sequences from Helicobacter pylori: lessons from Ladakh.. Proc Natl Acad Sci U S A.

[31] Falush D, Stephens M, Pritchard JK (2003). Inference of population structure using multilocus genotype data: linked loci and correlated allele frequencies.. Genetics.

[32] Kumar S, Tamura K, Nei M (2004). MEGA3: Integrated software for Molecular Evolutionary Genetics Analysis and sequence alignment.. Brief Bioinform.

[33] R (2006). Developement Core Team. R: A language and environment for statistical computing.. http://wwwr-projectorg.

[34] Martìnez H (2003). Determinación de las frecuencias de grupos sanguíneos y polimorfismos de ADN para dos estratos socioeconómicos de la población de Caracas..

[35] Acosta M (2002). Caracterizaciòn genética y composición étnica de la población de Churuguara, Edo. Falcòn, mediante los polimorfismos genéticos: ABO, Rh, VWA, FES/FPS y F13A01..

[36] Castro D (1993). Relación entre polimorfismos genéticos e historia en dos poblaciones negras venezolanas.. Bol Soc Esp Antrop Biol.

[37] Zambrano-Guzmàn O (1999). Estudio de la estructura genética de Hoyo de La Cumbre: un pueblo del Avila..

[38] Gonzàlez-Coira M, Mora J, Sepúlveda S, Cuevas J, Pérez J Evolución temporal “aparente” de la frecuencia génica de los sistemas ABO y Rh en una población de Mérida.; 1997..

[39] Castro-Guerra D, Zambrano-Guzmàn O (2000). Aporte génico español canario en tres poblaciones semiaisladas venezolanas; estimaciones hechas a partir de los sistemas ABO, Rh y -1-antitripsina.. Rev Esp Antrop Biol.

[40] Rodrìguez-Larralde A, Castro D, Gonzàlez-Coira M, Morales J (2001). Frecuencia génica y porcentaje de mezcla en diferentes àreas geográficas de Venezuela, de acuerdo a los grupos Rh y ABO.. Interciencia.

[41] Layrisse M, Wilbert J (1966). Indian Societies of Venezuela.; Instituto Caribe de Antropología y Sociología FlSdCN, editor.

[42] Arends T (1992). Estructura genética de la población indígena de Venezuela.

[43] http://www.bloodbook.com/world-abo.html (2007). Ratial and ethnic distribution of ABO blood types..

[44] Graven L, Passarino G, Semino O, Boursot P, Santachiara-Benerecetti S (1995). Evolutionary correlation between control region sequence and restriction polymorphisms in the mitochondrial genome of a large Senegalese Mandenka sample.. Mol Biol Evol.

[45] Plaza S, Calafell F, Helal A, Bouzerna N, Lefranc G (2003). Joining the pillars of Hercules: mtDNA sequences show multidirectional gene flow in the western Mediterranean.. Ann Hum Genet.

[46] Pinto F, Gonzalez AM, Hernandez M, Larruga JM, Cabrera VM (1996). Genetic relationship between the Canary Islanders and their African and Spanish ancestors inferred from mitochondrial DNA sequences.. Ann Hum Genet.

[47] Corte-Real HB, Macaulay VA, Richards MB, Hariti G, Issad MS (1996). Genetic diversity in the Iberian Peninsula determined from mitochondrial sequence analysis.. Ann Hum Genet.

[48] Crespillo M, Luque JA, Paredes M, Fernández R, Ramírez E (2000). Mitochondrial DNA sequences for 118 individuals from northeastern Spain.. Int J Legal Med.

[49] Picornell A, Gomez-Barbeito L, Tomas C, Castro JA, Ramon MM (2005). Mitochondrial DNA HVRI variation in Balearic populations.. Am J Phys Anthropol.

[50] Salas A, Comas D, Lareu MV, Bertranpetit J, Carracedo A (1998). mtDNA analysis of the Galician population: a genetic edge of European variation.. Eur J Hum Genet.

[51] Horai S, Murayama K, Hayasaka K, Matsubayashi S, Hattori Y (1996). mtDNA polymorphism in East Asian populations, with special reference to the peopling of Japan.. Am J Hum Genet.

[52] Torres MM, Bravi CM, Bortolini MC, Duque C, Callegari-Jaques S (2006). A Revertant of the Major Founder Native American Haplogroup C Common in Populations From Northern South America.. American Journal of Human Biology.

[53] Helgason A, Palsson G, Pedersen HS, Angulalik E, Gunnarsdottir ED (2006). mtDNA variation in Inuit populations of Greenland and Canada: migration history and population structure.. Am J Phys Anthropol.

[54] Alves-Silva J, da Silva Santos M, Guimarães P, Ferreira A, Bandelt HJ (2000). The Ancestry of Brazilian mtDNA Lineages.. Am J Hum Genet.

