# Supplementary figures and images for: Amerindian Helicobacter pylori Strains Go Extinct, as European Strains Expand Their Host Range
Source: PLoS One. 2008 Oct 2;3(10):e3307. doi: 10.1371/journal.pone.0003307 (PMC2551748; doi:10.1371/journal.pone.0003307)

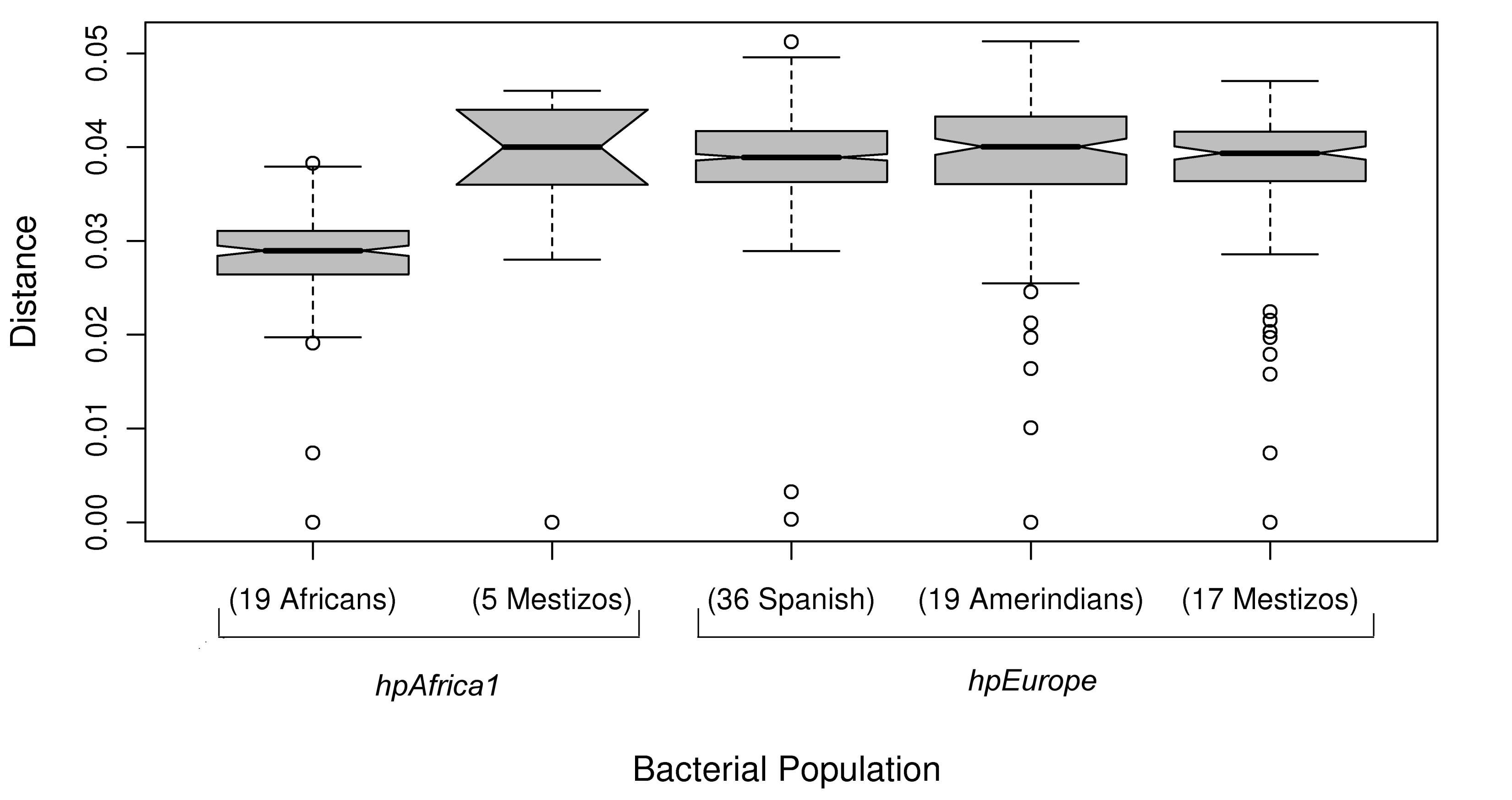

Supplement: Figure S1 — Intrapopulation genetic distance of H. pylori strains hpAfrica1 and hpEurope, by host source. hpAfrica1 strains from Mestizos were much more diverse than those from Africans (Kruskal-Wallis p<10−14). In contrast, neither Mestizos nor Amerindians increased the already high variability hpEurope strains from Spain. Medians are represented as the waist of the dress-like box, and the waist side openings indicate the 95% interval for the median. Above and below the median are the 3rd and 1st quartile respectively. The interval in dashed lines represents a maximum of 1.5× interquartile range and the open circles are outliers. Outliers are mostly low pairwise distances, indicating that similar pair of strains are less common than distant strains. (0.24 MB TIF) [file pone.0003307.s001.tif]

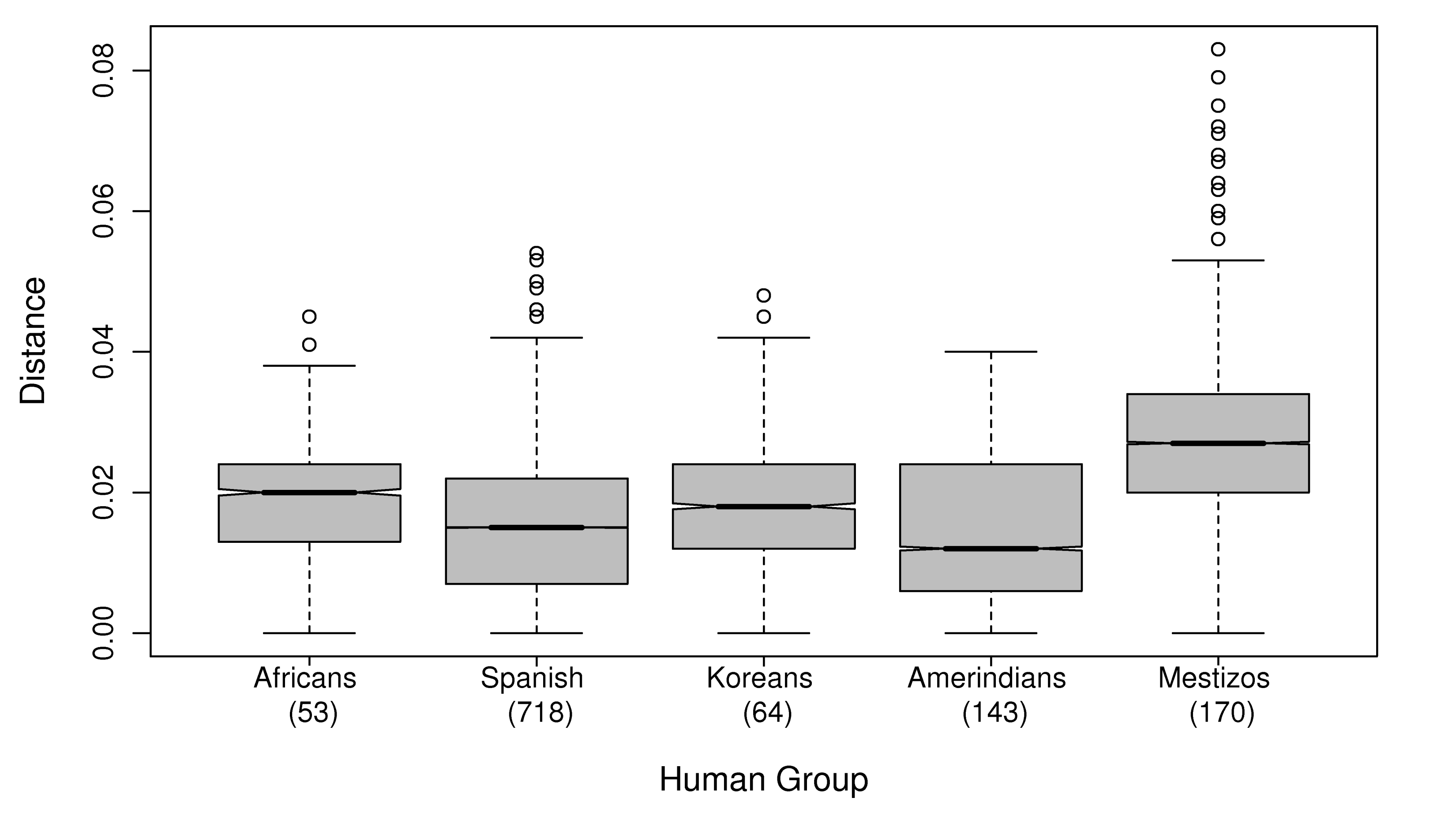

Supplement: Figure S2 — Genetic distances between mtDNA sequences of diverse human groups. Differences between median distances were significant for each of the human groups (Kruskal-Wallis test p<2.2×10^−15^; Wilcoxon with Bonferroni adjustment p<10−14). Permutation tests with 5,000 permutations confirmed human group differences (p = 0). Distances decreased in the order: mestizos>Africans>Koreans>Spanish> Amerindians. For explanation of the box plot see Figure S1. (18.39 MB DOC) [file pone.0003307.s002.doc]

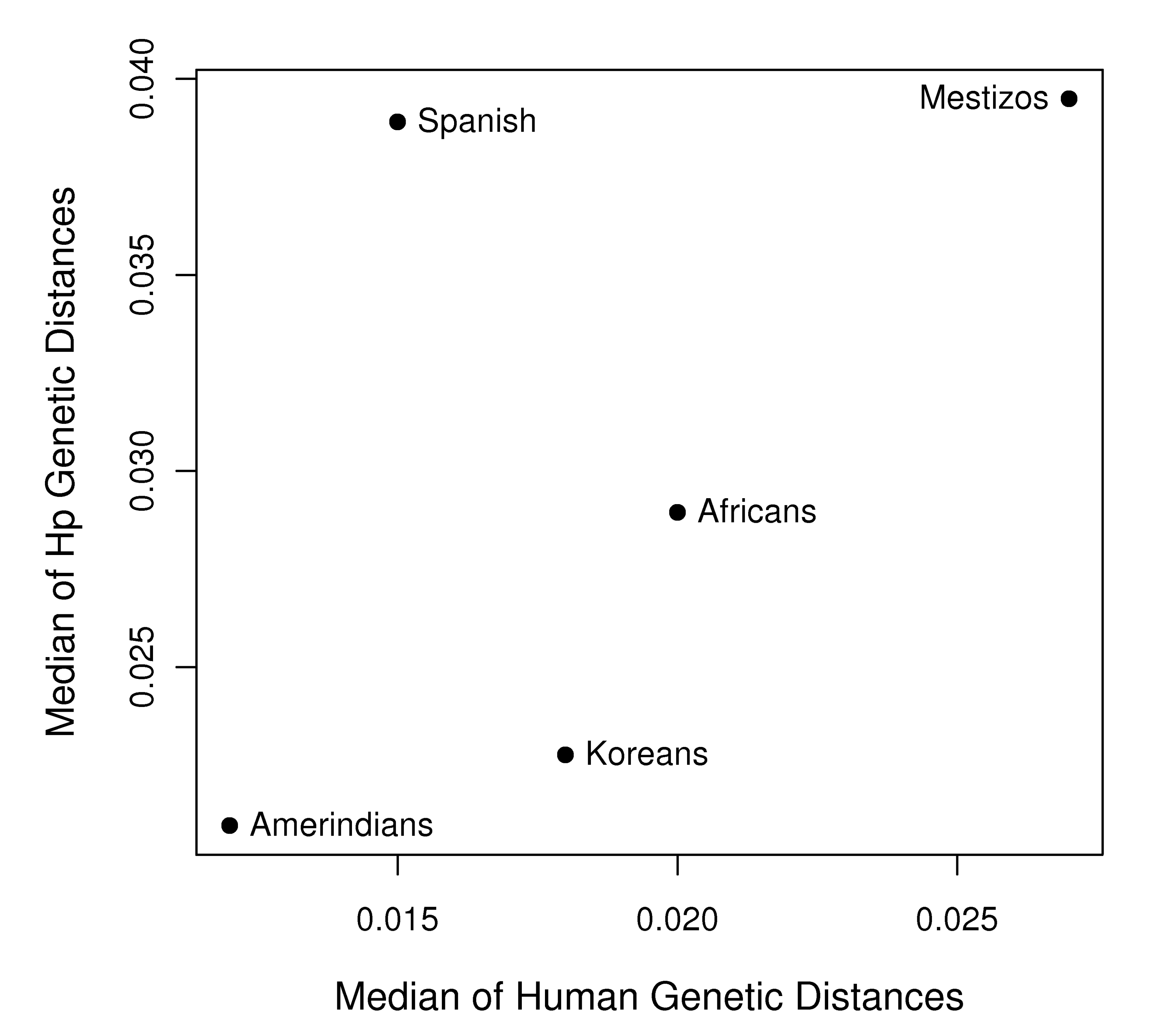

Supplement: Figure S3 — Genetic distances of human mtDNA sequences within Africans, Spanish, Koreans and groups of Amerindians and Mestizos. South American Amerindians studied have a degree of admixture as indicated by their higher genetic diversity than the Inuit. The Mestizos with African or Amerindian haplotypes have increased diversity in relation to Mestizos with European haplotypes. For explanation of the box plot see Figure S1. (0.26 MB TIF) [file pone.0003307.s003.tif]
